# Supplementary material for: Association of Health-Related Quality of Life with Overall Survival in Older Americans with Kidney Cancer: A Population-Based Cohort Study
Source: Healthcare (Basel). 2021 Oct 10;9(10):1344. doi: 10.3390/healthcare9101344 (PMC8544450; doi:10.3390/healthcare9101344)
Supplement: Supplementary file 1 [file healthcare-09-01344-s001.zip › healthcare-1391184 SUPPLEMENTARY NEED TO UPDATE.pdf]

Supplementary Material

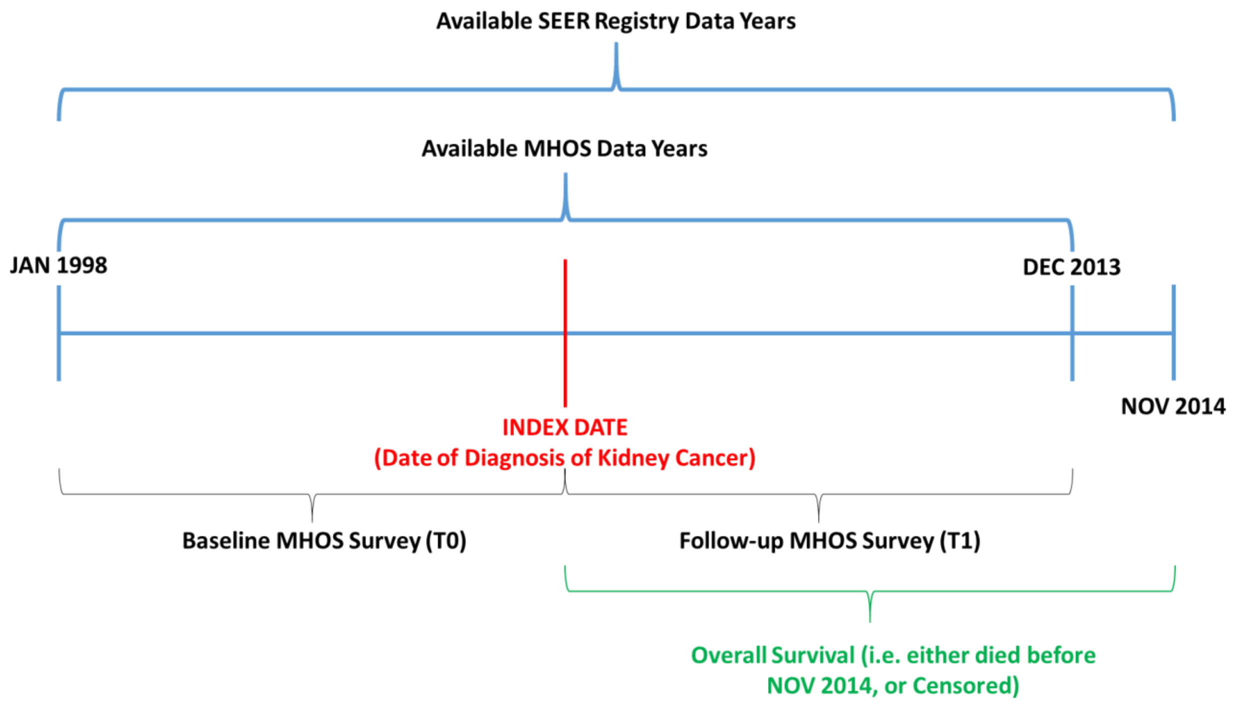

Supplementary Figure S1. Graphical Representation of Study Design

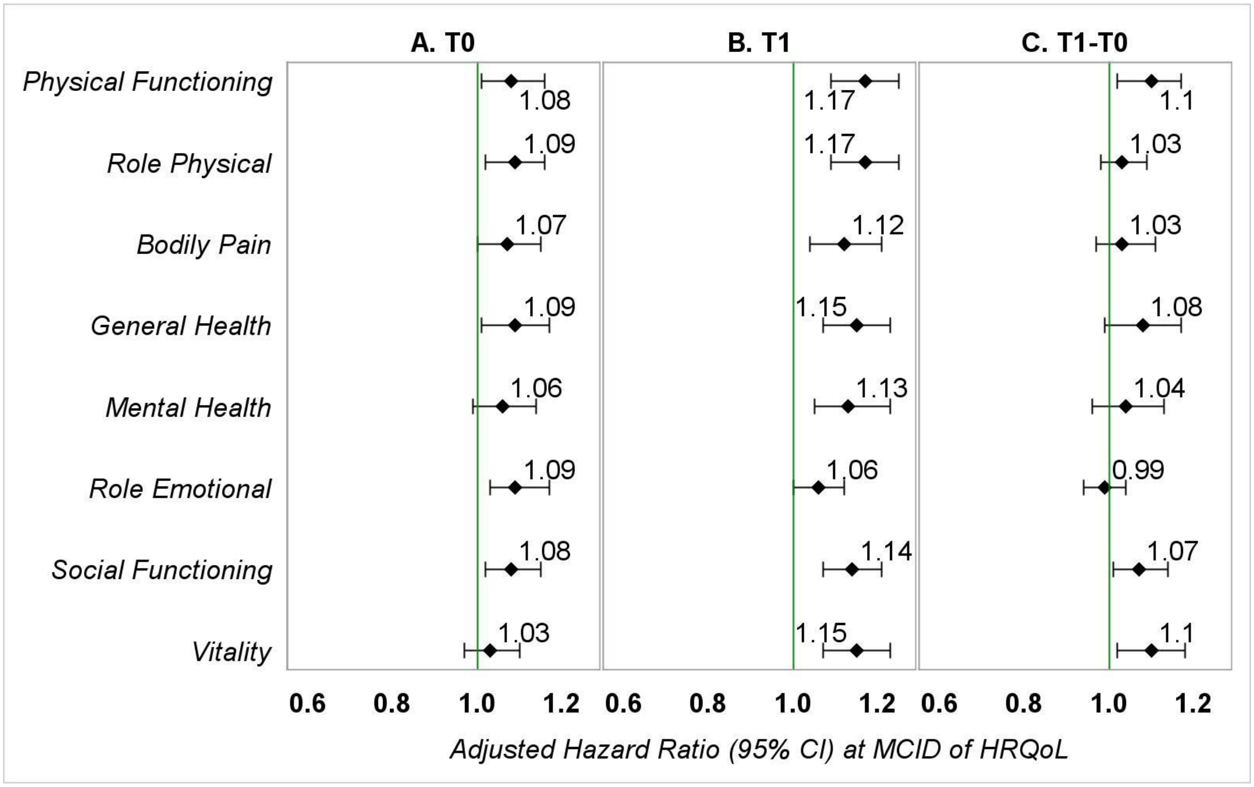

Supplementary Figure S2. Adjusted Hazard Ratios (95% CI) Representing the Association between HRQoL Scales (at MCID) and OS in Patients with Kidney Cancer – ***Main Analyses***

Legend

A. Hazard ratios represent the association between *baseline HRQoL scales (3-point reduction) and overall survival* adjusted for gender, race, education (T0), marital status (T0), annual household income (T0), smoking status (T0), geographic region, number of comorbid conditions (T0), age at diagnosis, stage of kidney cancer (KC) at diagnosis, tumor grade, treatment type, and months between the T0 Survey and KC diagnosis.
B. Hazard ratios represent the association between *follow-up HRQoL scales (3-point reduction) and overall survival* adjusted for gender, race, education (T1), marital status (T1), annual household income (T1), smoking status (T1), geographic region, number of comorbid conditions (T1), age at diagnosis, stage of kidney cancer (KC) at diagnosis, tumor grade, treatment type, and months between diagnosis of KC and the T1 Survey.
C. Hazard ratios represent the association between *change in HRQoL scales (3-point reduction) from baseline to follow-up and overall survival* adjusted for gender, race, education (T1), marital status (T1), annual household income (T1), smoking status (T1), geographic region, number of comorbid conditions (T1), age at diagnosis, stage of kidney cancer (KC) at diagnosis, tumor grade, treatment type, months between the T0 Survey and KC diagnosis, and months between diagnosis of KC and the T1 Survey.

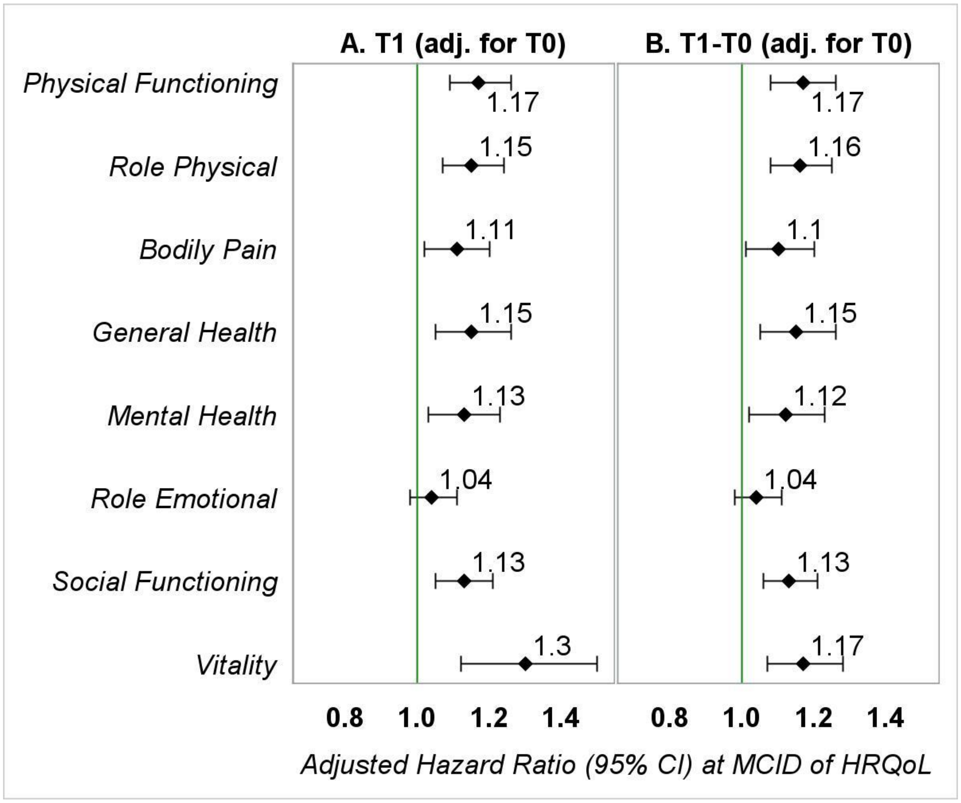

Supplementary Figure S3. Adjusted Hazard Ratios (95% CI) Representing the Association between HRQoL Scales (at MCID) and OS in Patients with Kidney Cancer – *Sensitivity Analyses I*

Legend

A. Hazard ratios represent the association between *follow-up HRQoL scales (3-point reduction) and overall survival* adjusted for gender, race, education (T1), marital status (T1), annual household income (T1), smoking status (T1), geographic region, number of comorbid conditions (T1), age at diagnosis, stage of kidney cancer (KC) at diagnosis, tumor grade, treatment type, months between diagnosis of KC and the T1 Survey, and baseline HRQoL
B. Hazard ratios represent the association between *change in HRQoL scales (3-point reduction) from baseline to follow-up and overall survival* adjusted for gender, race, education (T1), marital status (T1), annual household income (T1), smoking status (T1), geographic region, number of comorbid conditions (T1), age at diagnosis, stage of kidney cancer (KC) at diagnosis, tumor grade, treatment type, months between the T0 Survey and KC diagnosis, months between diagnosis of KC and the T1 Survey, and baseline HRQoL.

Supplementary Table S1. Health-related Quality of Life of Kidney Cancer Patients

HRQoL	Total (N=188), mean ± SD	Group, mean ± SD		P-value
		Survived (n=99, 52.7%)	Died (n=89, 47.3%)	
**Baseline (T0)**				
Health Utility (SF6D/VR6D)	0.70 ± 0.13	0.71 ± 0.13	0.69 ± 0.13	0.227
Physical Component Summary	38.2 ± 12.2	39.3 ± 12.7	36.9 ± 11.5	0.179
Mental Component Summary	52.9 ± 10.4	54.8 ± 9.0	50.8 ± 11.4	0.009
Physical Functioning	39.0 ± 13.1	39.9 ± 13.6	38.0 ± 12.4	0.323
Role-physical	41.2 ± 12.7	42.6 ± 12.9	39.7 ± 12.5	0.120
Bodily Pain	42.3 ± 11.8	43.5 ± 11.2	40.9 ± 12.4	0.136
General Health	44.5 ± 11.0	45.6 ± 10.8	43.2 ± 11.2	0.136
Mental Health	51.4 ± 10.5	52.4 ± 10.4	50.3 ± 10.5	0.177
Role-emotional	49.2 ± 11.2	51.1 ± 9.6	47.1 ± 12.5	0.017
Social Functioning	47.1 ± 12.5	49.4 ± 11.9	44.7 ± 12.7	0.009
Vitality	42.3 ± 10.9	48.7 ± 10.9	45.7 ± 10.7	0.056
**Follow-up (T1)**				
Health Utility (SF6D/VR6D)	0.65 ± 0.12	0.69 ± 0.12	0.62 ± 0.12	<0.001
Physical Component Summary	34.0 ± 12.0	36.9 ± 12.1	30.7 ± 11.2	<0.001
Mental Component Summary	50.6 ± 11.6	53.1 ± 10.6	47.8 ± 12.1	0.002
Physical Functioning	34.5 ± 13.7	36.6 ± 14.1	32.1 ± 12.8	0.024
Role-physical	38.1 ± 12.4	41.9 ± 12.6	33.9 ± 10.8	<0.001
Bodily Pain	40.6 ± 11.8	42.4 ± 11.3	38.5 ± 12.0	0.025
General Health	39.1 ± 11.3	41.8 ± 10.9	36.1 ± 11.0	<0.001
Mental Health	49.3 ± 10.9	51.3 ± 10.7	47.1 ± 10.7	0.009
Role-emotional	45.8 ± 13.1	48.1 ± 11.9	43.2 ± 13.9	0.012
Social Functioning	43.2 ± 13.6	47.0 ± 12.5	39.0 ± 13.7	<0.001
Vitality	43.2 ± 11.4	45.9 ± 10.8	40.2 ± 11.3	<0.001
**Change from T0 to T1**				

Health Utility (SF6D/VR6D)	-0.05 ± 0.11	-0.03 ± 0.11	-0.07 ± 0.11	0.023
Physical Component Summary	-4.1 ± 11.0	-2.4 ± 11.4	-6.1 ± 10.2	0.023
Mental Component Summary	-2.4 ± 11.8	-1.7 ± 11.7	-3.1 ± 12.0	0.450
Physical Functioning	-4.6 ± 12.7	-3.3 ± 12.3	-6.0 ± 13.1	0.157
Role-physical	-2.9 ± 13.7	-0.5 ± 13.2	-5.6 ± 13.8	0.011
Bodily Pain	-1.6 ± 11.0	-0.9 ± 11.5	-2.3 ± 10.4	0.387
General Health	-5.0 ± 10.5	-3.3 ± 9.5	-6.9 ± 11.3	0.024
Mental Health	-2.1 ± 11.5	-1.2 ± 11.3	-3.0 ± 11.8	0.275
Role-emotional	-3.4 ± 14.9	-3.0 ± 14.1	-3.9 ± 15.8	0.670
Social Functioning	-3.9 ± 12.9	-2.5 ± 12.6	-5.5 ± 13.2	0.107
Vitality	-3.9 ± 10.8	-2.8 ± 11.6	-5.1 ± 9.9	0.152

Supplementary Table S2. Adjusted Hazard Ratios (95% CI) Representing the Association between HRQoL (at MCID) and OS in Patients with Kidney Cancer – *Sensitivity Analyses II/III*

Measures of HRQoL	**T0 Cohort (N=1,055)** *SA-II*	**T1 Cohort (N=966)** *SA-III*
Health Utility (SF6D/VR6D)	1.02 (1.00, 1.05)	1.10 (1.07, 1.13)
Physical Component Summary	1.04 (1.01, 1.06)	1.10 (1.07, 1.13)
Mental Component Summary	1.01 (0.99, 1.04)	1.07 (1.04, 1.09)
Physical Functioning	1.01 (0.99, 1.03)	1.08 (1.06, 1.11)
Role-physical	1.02 (1.00, 1.04)	1.08 (1.05, 1.10)
Bodily Pain	1.02 (0.99, 1.04)	1.05 (1.02, 1.08)
General Health	1.03 (1.00, 1.05)	1.08 (1.06, 1.11)
Mental Health	1.01 (0.98, 1.03)	1.05 (1.03, 1.08)
Role-emotional	1.00 (0.98, 1.02)	1.05 (1.02, 1.07)
Social Functioning	1.03 (1.01, 1.06)	1.09 (1.07, 1.12)
Vitality	1.01 (0.99, 1.04)	1.09 (1.06, 1.12)

Point estimates highlighted in grey indicate deviation from the respective estimates in the main analyses in regards to statistical significance
